# Whose Elimination? Frontline Workers’ Perspectives on the Elimination of the Human African Trypanosomiasis and Its Anticipated Consequences

**DOI:** 10.3390/tropicalmed5010006

**Published:** 2020-01-01

**Authors:** Jean-Benoît Falisse, Erick Mwamba-Miaka, Alain Mpanya

**Affiliations:** 1 Centre of African Studies, University of Edinburgh, Edinburgh EH8 9LD, UK; 2 Programme National de lutte contre la THA (PNLTHA), Kinshasa 2, Democratic Republic of the Congo; erickmwamb2002@yahoo.fr (E.M.-M.); mpanya_alain@yahoo.fr (A.M.); 3 Faculté des Sciences de la Santé, Université Pedagogique Nationale (UPN), Kinshasa BP 8815, Democratic Republic of the Congo

**Keywords:** Human African Trypanosomiasis (HAT), disease elimination, disease eradication, frontline workers, DR Congo, mobile screening, qualitative methods

## Abstract

While academic literature has paid careful attention to the technological efforts―drugs, tests, and tools for vector control―deployed to eliminate Gambiense Human African Trypanosomiasis (HAT), the human resources and health systems dimensions of elimination are less documented. This paper analyses the perspectives and experiences of frontline nurses, technicians, and coordinators who work for the HAT programme in the former province of Bandundu in the Democratic Republic of the Congo, at the epidemic’s very heart. The research is based on 21 semi-structured interviews conducted with frontline workers in February 2018. The results highlight distinctive HAT careers as well as social elevation through specialised work. Frontline workers are concerned about changes in active screening strategies and the continued existence of the vector, which lead them to question the possibility of imminent elimination. Managers seem to anticipate a post-HAT situation and prepare for the employment of their staff; most workers see their future relatively confidently, as re-allocated to non-vertical units. The findings suggest concrete pathways for improving the effectiveness of elimination efforts: improving active screening through renewed engagements with local leaders, conceptualising horizontal integration in terms of human resources mobility, and investing more in detection and treatment activities (besides innovation).

## 1. Introduction

Gambiense Human African Trypanosomiasis (HAT), also known as sleeping sickness, is a parasitic tropical infectious disease whose main vector of transmission is the tsetse fly. It is one of the 17 neglected tropical diseases that the World Health Organisation (WHO) put on its 2012 agenda of diseases to be eliminated as a public health problem by 2020, with the additional target of stopping all HAT transmissions by 2030 [1,2,3,4]. The abundant literature that has accompanied HAT elimination efforts is primarily focussed on technological innovations, including the development of new drugs, tests, and tools for vector control [4,5,6,7]. Only limited attention has been paid to the frontline human and health systems sides of the effort [8,9,10,11]. The present article looks at the experiences and perspectives of frontline workers (nurses, doctors, lab technicians and assistants, and clinic and provincial managers) involved in HAT screening and treatment at a time marked by optimistic discourses about and concrete achievements towards the elimination of a disease many frontline workers have dedicated their lives to combatting.

The study is set in the Democratic Republic of the Congo (DR Congo), a country that reportedly sees 80% of the new HAT cases and counts 63% of the at-risk population [12], and revolves around two research questions. The first is: how does elimination look like from the perspective of those doing it in the field? Epidemiologists see the curve of HAT cases dropping, and clinical researchers celebrate more straightforward and more efficient drugs and tests, but what does it mean for those at the frontline? Do they agree that the epidemic is decreasing and the tools are good? Do they think HAT will be eliminated soon? Frontline workers’ viewpoints and experiences matter because they are the embodied expression of national and global programmes. The second question relates to the anticipation of life and livelihood ‘after the end of the disease’ [13,14]: how does elimination affect the prospects of HAT workers? In DR Congo, the fight against HAT is through a vertical programme made of specialised health workers who have often spent their entire career specialising on HAT [15]; HAT elimination would undoubtedly change their work. Do they feel insecure or confident about the future? We believe that these behavioural and organisational aspects are fundamental to comprehend as they can positively or negatively influence the provision of HAT screening and treatment services and are essential to HAT elimination efforts, especially when bearing in mind that periods of low incidence can be vulnerable to a resurgence of the disease (as control efforts ebb away). Our research is also important to help governments prepare their systems for a post-elimination future.

The next section briefly discusses the situation of HAT elimination in DR Congo and explains the institutions and instruments deployed in the field. The methods are then presented; they build on a series of interviews with frontline workers. The results are divided into three sub-sections. The first provides (1) details on HAT careers and the nature of frontline work while the remaining two correspond to the two research questions: one looks at (2) frontline workers perspectives on HAT elimination and the other at (3) perspectives on HAT workers’ future. A discussion section puts the results in perspective with the literature on HAT elimination and human resources in the context of disease elimination in general.

## 2. HAT Elimination in DR Congo

In DR Congo, as in other central African countries, two major HAT epidemics took place during the twentieth century. The first began in 1900 and peaked in the 1930s with over 30,000 new cases annually [15]; it was brought under control by 1960, following the organisation of mass screening campaigns with mobile teams made up of specialised nurses and lab technicians that visited villages and other sites at risk [4,16]. Prevalence levels then reached one case per 10,000 people, and it was believed that the disease would stop spreading [12]. It was an erroneous assumption: as the country entered its turbulent post-independence era and control was somewhat diminished, the number of cases increased again [17]. By 1998, there were 26,318 new cases per year [15].

Over the past twenty years, though, through the efforts of the national HAT control programme, the *Programme National de Lutte contre la Trypanosomiase Humaine Africaine* (PNLTHA), non-governmental organisations, and donors, the number of HAT cases has been reduced to a mere 660 new cases in 2018 [18]. This reduction is readily associated with new technology including (door-to-door) screening tools, treatment, vector control methods, and data collection and data analysis systems [12,19,20]. As in other parts of the world, international actors and the government of DR Congo have embraced the 2020 target, and on 31 January 2018, the government launched the first National HAT Day to celebrate its commitment to the goal. At this occasion, it outlined a new strategy of elimination through “new technologies and an innovative approach to early screening for the disease” [21].

The success of HAT case reduction in DR Congo may, however, come with drawbacks. First, fewer cases may be associated with a demotivation of populations in endemic areas to participate in mass screening campaigns, as HAT is no longer perceived as a threat. Second, rarer cases also affect the health system put in place as there is a risk that staff and funders change interest. The evidence is mostly anecdotal (at this stage), but it invites studies like ours to be careful.

HAT control and treatment in the DR Congo is organised by the PNLTHA, which sits as a vertical programme within the Ministry of Health. The centralisation of expertise inside a vertical programme is possibly stronger than in other countries owing to historical legacies but also the spread of the epidemic that has made HAT careers a reality in the country. The PNLTHA is reliant on external funding for substantial parts of its activities, which does at times put pressure on frontline staff. Besides the vertical programme, which constitutes the bulk of the effort, and similarly to many other countries affected by sleeping sickness, some non-specialised health facilities have integrated HAT-related activities. In this case, the frontline workers would typically have many other responsibilities beyond HAT screening and treatment. The present paper focusses on PLNTHA workers.

Control strategies consist of active early detection, passive detection, and treatment of patients, which are complemented by vector control activities. Early detection through active screening is provided by mobile teams of the PNLTHA. A mobile team consists of 7 to 9 people who travel by vehicle to endemic villages to organise mass screening campaigns. A mobile team has the capacity to examine around 66,000 people per year [22]. There are currently 30 mobile teams and 18 mini-teams on motorbikes, with the latter corresponding to a recent evolution in the mass screening approach [18]. The recent years have also seen new micro-planification strategies with dedicated software to focus efforts according to the recorded prevalence of HAT. Premiums, a form of pay-for-performance, are paid to the mobile teams to incentivise them to identify cases and cover vast areas. The activities of the mobile teams are complemented by passive surveillance with so-called ‘fixed’ health facilities that are either solely focussed on HAT or include HAT treatment and screening as a part of a broader package of activities. As shown by Mitashi et al. in a 2015 study, it is important to note that the possibility of extending passive screening to all primary healthcare centres is not straightforward: such health facilities typically lack the tools and ability to diagnose HAT [23]. A more recent study by Mulenga et al. is only slightly more optimistic about the potential of passive screening at a wider scale [24]. This has constituted an impetus to the development of new, easier, diagnostic and treatment tools meant to facilitate the integration of HAT activities in endemic areas [5].

HAT screening can be syndromic or serological. Syndromic screening is often late because the signs and symptoms of HAT are not specific enough to allow easy clinical diagnosis of the disease [25,26]. Syndromic screening is typically based on signs of the second stage of the disease and therefore leads to late diagnosis. Serological screening is done on the basis of an agglutination test for Trypanosomiasis (CATT), which is commonly used in mass screening by mobile teams [27]. Significant progress has been made in recent years with the development of a new rapid diagnostic test (RDT) that is individual and easy to use. Patients diagnosed by mobile teams are referred for treatment in health facilities.

The treatment of HAT is stage-dependent. In the first stage, Pentamidine, which appeared in 1940, is still used today [28]. For the second stage, the Nufurtimox-Eflornithine (NECT) combination therapy [29,30] has replaced Melarsopol since 2009. Given the volume of cases it hosts, DR Congo is the focus of ongoing trials and projects to develop HAT innovations that will shape the course of elimination domestically as well as in neighbouring countries. They include two oral drugs, Fexinidazole, which was approved by the Ministry of Health and will soon be used across the country, and Acoziborole.

Finally, HAT control in the field also consists of vector control, which is a complementary strategy to the screening and treatment of patients and is also coordinated by the PNLTHA. The newest technology in this field is the ‘tiny targets’: small insecticide-impregnated screens that are cheap and easy to use and can be deployed on a large scale [31].

## 3. Materials and Methods

To understand the perspectives of frontline HAT providers, we conducted semi-structured interviews with officers of the national vertical programme (PNLTHA) who work at the intermediate and peripheral levels of HAT control in the former province of Bandundu. Bandundu has hosted around 50% of all patients diagnosed with HAT in recent years [32,33]. At the time of the interviews in February 2018, HAT screening and treatment activities were carried out by 13 mobile teams doing mass screening in endemic HAT villages, 18 mini-teams that organised home-based HAT screening, 20 ‘fixed’ health facilities specialised in HAT screening and treatment (*Centre de Dépistage, Traitement et Contrôle de la THA* (CDTC)) and several other health centres and general reference hospitals that had integrated passive screening and treatment of patients into their package of activities. Two provincial HAT coordinators (North Bandundu and South Bandundu) were in charge of the mobile teams and treatment and screening centres. The province of Bandundu is now divided into three separate provinces: Kwilu (our fieldwork sites: Bandundu ville, Kikwit, Masinanimba, Bagata, and Yasa Bonga), Maidombe (our fieldwork site: Mushi) and Kwango (our fieldwork site: Kenge). 

The research participants worked at the levels of the mobile teams (team leader, secretary, and laboratory technician), at the health facilities that specialise in HAT (nurse in charge of the centre) and at the provincial HAT coordination (coordinating physician and provincial supervisor). It is important to note that our study is only about PLNTHA workers, who are the main responders to the HAT epidemic (but not the only ones, as explained earlier). Sampling was oriented to include participants of different ages and profiles, and the interviews were organised until saturation, which was reached with a total of 21 interviews. Table 1 sums up the profile of the interviewees and includes the interview number that is referenced in the analysis.

The interviews were led by the Congolese co-author (Mpanya) who was already known to some of the interviews (as he has been conducting research in this field for years). Participation was voluntary, and participants were invited for interviews at an agreed location according to their availability. The interviews were conducted in the local language, in Lingala, or in French, depending on the choice of the interviewee. They were recorded on a digital medium with the consent of the interviewee and lasted on average 35 min (the longest took 56 min). The recordings were transcribed and entered in MS Word, and the data were coded and analysed using Nvivo (12.0).

An interview guide was used and is available in Appendix A. The main themes of the guide were directed by our research questions and focussed on the respondents explaining (1) their career in HAT control, (2) the perception of the current efforts vis-à-vis elimination (number of cases, elimination objectives, etc.), and (3) how they perceive their life will be after HAT elimination (if conceivable to them at all). There was no apparent risk to study participants, and the study protocol was approved by the Ethics Committee of the Faculty of Medicine of the University of Lubumbashi and the School of Social and Political Science at the University of Edinburgh. The anonymity and confidentiality of the participants were guaranteed. The consent of the study participants was obtained orally and recorded; written consent would make participants uncomfortable to express themselves easily. Only the two researchers who conducted this research and the assistant who helped with transcription have had access to the collected information.

## 4. Results

A series of key themes emerged through the coding and analysis of the interviews: many are directly derived from our interview guide, but others were unanticipated such as HAT ‘vocations’, the preoccupations with vector control, or the expectation that the state will provide future employment after HAT elimination. We identified a total of 12 (key) interrelated topics, which we will present through three sub-section. The first sub-section characterises the profiles, careers, and livelihoods of those involved in frontline HAT control; it shows entire lives devoted to fighting the disease and work conditions that are improving on some aspects and deteriorating on others. The second sub-section shows how HAT workers perceived the announced imminent elimination of the disease; most remain careful and point to two challenges: the coverage of HAT screening activities and difficulties of eliminating the vector. The third sub-section documents the ways in which HAT workers see their lives after elimination and how they are preparing for it.

### 4.1. Work at the Frontline

Two elements appear central to understanding the experience of frontline workers: the changing nature of their work in recent years and their keen sense of attachment to HAT control.

As touched upon in the background section, HAT control technology has evolved substantially over recent decades. It has deeply affected the work done at the frontline. On the one hand, all our interviewees recognise that new technology such as RDTs and NECT has rendered their work easier. On the other hand, though, the sharp decrease in the number of cases in the last two decades has also had a clear financial and organisational impact on work, especially for mobile units.

In financial terms, fewer cases have meant fewer premiums paid to team members for detecting positive cases. The interviews led to ample discussions about the workers’ pay, with mobile unit members complaining that their income has diminished in real terms because of the increased cost of life and the suppression or impossibility to obtain premiums beyond the monthly ‘fixed’ (*prime fixe*) and ‘mobility’ (*prime d’itinérance*, premium for being in a mobile team) premiums. For a regular mobile team member, the former is around $100, while the latter is around $200.

The main effect of the progressive elimination of the disease is, however, in terms of workload. Fewer cases typically mean more time spent looking for cases. Mobile teams are asked to spend more extended periods of time in the field. Twenty days per month, which can extend to 22 or 24 days, are the norm. While mobile unit members explain that they are used to spending time in the field, they also explain that 20-day missions lead to complications with family lives and relationships: “it penalises our wives and children” explains a mobile unit member (1, mobile unit). Longer distances also mean that it is harder to come back urgently, with workers reporting cases of colleagues being unable to attend dying partners and children.

Another unanimously reported element that has made work harder is the changing socio-political environment, with the local leaders (customary chiefs, community notables, and community health workers) less inclined to help the teams, sometimes asking to be paid to provide assistance. This is a change from the time when mobile units could automatically count on the support of the chiefs, as explained by a mobile unit veteran:

“in the time of Mobutu [in the 1970s?] the authorities would fine those who did not come to us, so people came en masse, as they feared to be arrested. But now, the authorities don’t do that. […] now it is difficult to get people as the authorities do not get involved”

(8, mobile unit)

Against this changing nature of work, a second critical element that stands out in the HAT frontline workers’ narratives of their careers is their distinctive attachment to the cause of fighting HAT and to the programme they serve, the PNLTHA. This attachment builds on several entangled elements: long careers, ‘vocations’ for HAT, comradery and a sense of belonging to a community, and the conviction of being involved in a noble project.

Our study is not a systematic survey of HAT workers, but it is clear from the interviews that many on the frontline spent most if not all of their careers in HAT. Our interviewees often have decades of experience, which is not a coincidence or a sampling anomaly; rather, it signals that careers are being made in HAT. As explained by our participants, when people start working on HAT, they often keep working on HAT. All the mobile unit leaders climbed up the ranks, and so did the coordinators of the treatment centres. The oldest interviewees call themselves the ‘second generation’ (8, mobile unit) as they came after the first generation of mobile units and dispensaries set up under Belgian rule. Long careers create a sense of attachment and a strong identification with HAT control efforts that are described as a core dimension of people’s lives, as one senior interviewee explained:

“When I started working, I was not married yet. I married ‘in’ [*dans*] sleeping sickness, I got children in sleeping sickness, I schooled my children in sleeping sickness, I built [my house] in sleeping sickness. I could say a good part of my life is built on sleeping sickness”

(15, frontline non-mobile)

In around a third of the cases, the interviewees referred to a vocation for HAT control. The origins of such vocation were explained as typically revolving around the traumatic experience of witnessing a patient or a relative dying of HAT or its treatment with Arsobal (*melaprosol*), or around a charismatic mentor. Well-known figures of HAT control in the DR Congo such as Constantin Miaka (former director of PLNTHA) or Herman Bruneel (head of FOMETRO, an NGO that supported the fight against HAT in the 1980s and 1990s) as well as less well-known HAT workers―including, in one case, the father of the interviewed HAT worker―are cited as mentors by workers at all levels of responsibility.

Such a sense of closeness between frontline actors and national level figures such as Miaka naturally leads to discussing another key feature of HAT work: the sense of belonging to a strong community that is clear from all interviews. Such a strong bond is expected in the case of the mobile unit members that spend extensive amounts of time together in hard conditions, but it was also depicted at higher levels of responsibility, at the provincial level (as well as the national level, in a related piece of research we conducted on national level actors). The HAT community, which many described using the word ‘family’, is primarily but not solely Congolese and includes national and foreign researchers, whom one respondent describes as part of a virtuous circle of information sharing and learning. A senior nurse explained the importance of this community:

“[what I like most about HAT is] the family of the fight against Trypanosomiasis that we have been able to have. Wherever we went, we met people who are in this fight; it really gives us joy; it’s really great memory for me, unforgettable because we met a lot of people, of all categories and even some people from foreign countries”

(18, management)

Ultimately, this sense of attachment also relates to the idea that HAT workers have of themselves as a specialised unit, which has given them the opportunity to obtain a higher social rank. While staff members regularly complain about their pay, and possibly rightly so, they remain better paid than both public health workers at the same level of qualification and the average Congolese (GDP per capita was $457.58 in 2017), and perhaps more so than in the past as the general job market has deteriorated, as reported by a coordinator (19, management). The sense of HAT control as a form of ‘social ladder’ is especially visible among older workers of mobile units. When asked what their best memory of HAT control is, two of them spontaneously explained:

“my children have studied, my eldest will soon become a doctor, he is studying in Kinshasa […]. I have also bought a plot of land here in Bandundu.”

(20, mobile unit)

“well, I can say that with the little they give me at the end of the month, that’s what allows me to have my children study […], so, in short, my children study.”

(2, mobile unit)

A third one explained that the benefit is not only financial. As a doctor, “it was through sleeping sickness that I was able to get this reputation [*heritage*], and sleeping sickness made my name known” (9, management).

Fully understanding that work at the frontline remains hard, albeit in new ways, is crucial to mobilise efforts to achieve HAT elimination. This sub-section has also highlighted the distinctive identity of HAT workers who are characterised by a strong sense of community and common cause and whose professional activity has been, especially for the least qualified, akin to social elevation.

### 4.2. Perspectives on Elimination

Almost all the interviewees agree that the epidemiological decrease claimed at the national level rings true with their observations as frontline workers―the epidemic is going down. They associate such trends with the efforts of the PNLTHA (but rarely directly cite new technology), and some also identify more systemic change that affects the vector, such as new land use:

“The large forests where tsetse flies used to hide are being pulled up, people are starting to cultivate the fields there, they are making small ponds; and even here in the village, there are no tsetse flies left.”

(14, frontline non-mobile)

Importantly and interestingly, though, none of the 21 interviewees felt totally comfortable with the year 2020 being selected as the point of elimination of HAT as a public health problem. As we will discuss later, this view contrasts with the view of international and national public health policymakers. The most optimistic of our respondents, around a third of the sample, foresaw the end of the epidemic somewhere between 2022 and 2025, provided that substantial resources go to control. The rest of the respondents were cautiously optimistic; they saw the epidemic as “nearing the end”, but they would not risk committing to an end date. Half the respondents did not appear to believe that total elimination was possible; rather they compared the future of HAT to the presence of diseases that have now become very rare in DR Congo, such as leprosy:

“Sleeping sickness can be eliminated, but it will be like leprosy. [They told us it is eliminated but] I saw the people here in Bandundu who don’t have toes. When I asked the question, I was told that there was a disease called leprosy, Maba’s disease. Even today, we, the mobile unit, have friends who are on the leprosy and tuberculosis programme.”

(1, mobile unit)

The reason frontline workers are not fully confident that elimination will happen in the short run is related to two aspects: the coverage of their control activities and the work on vectors. It is important to note that none of them questioned the technology. There is no doubt, in the mind of the interviewees, that the tools at their disposal―first and foremost RDT and NECT―are good. However, they do not see further technological progress as absolutely necessary for future progress. The only exception is one interviewee who stressed that finding a way that allows avoiding lumbar punctures would be important (he was apparently unaware of the Fexinidazole trial). Rather, as the two quotes below illustrate, the issue is with the organisation and efficiency of control and sensitisation activities that enable frontline workers to put technology into use:

“to eliminate the disease, two problems need to be solved. First of all, on the molecular side, we have succeeded, we have molecules that are really good. [...] the treatment [NECT] is not as toxic as before [Arsobal]. [The other side is] our explanations in the villages [...] there will be no elimination until everyone understands [can identify] sleeping sickness.”

(8, mobile unit)

“It is more a question of organisation than just tools. The tools may be there, but if they are not properly used or if the conditions of use of these tools are not met, elimination will not be achieved.”

(10, management)

The question of the sensitisation of the population is twofold. One issue, as expressed in the quotes above and as alluded to earlier has to do with the difficulty of convincing the population and their leaders to get tested and to report suspicious cases. This is partly a problem of knowledge (people cannot identify the disease) but also a broader issue of trust in the health system:

“Up to now our population, the whole of our population, is not sensitive to active screening. They have their reasons. There are some people who say that sleeping sickness is sometimes transmitted by us, the agents, by evil spirits.”

(7, mobile unit)

It is also a wider issue of trust in politico-administrative authorities, which echoes the deeper problems of governance in the endemic areas:

“The political-administrative authorities [likely referring to the leaders mentioned earlier] are not so much listened to by the population. […] The population trusts us, but I tell you, the population no longer really trusts the agents of the state [government and administrators], but we greatly need them because the population belongs to them, we must go through their channel to try to take care of their population.”

(5, mobile unit)

This issue has led, as hinted at earlier, to giving monetary incentives to community leaders. It is seen as tricky by frontline workers who both criticise the situation but also find themselves with no other option than asking the programme and funders to give them such money. This is a change, “in the old days when we went to a village; we gave nothing to the elders” (3, mobile unit) explains a nurse. Another explains how they now have to:

“give people a motivation [understood as a financial incentive in this context] to access people in the villages where we go, because we are dealing with village chiefs, elders, religious leaders and community relays, they are the ones who help us to do our job well. Motivation is one of the realities we encounter in the field in villages”

(2, mobile unit)

The question of community involvement and sensitisation, combined with the coverage of active screening activities, leads to the core argument of the frontline workers for why elimination may be farther away than expected: the low level of coverage of the territory by PNLTHA activities. Almost all research participants expressed this concern; some even explained that in their view, such low coverage might hide the real size of the epidemic:

“The decrease in [reported] cases, here I would just like to explain that it is not necessarily related to [a true] decrease in endemicity. So endemicity may still exist, but since the level of control has decreased due to lack of resources, it may also explain the decrease in the number of cases.”

(18, management)

While many understand that the areas covered are the most at risk of harbouring disease, they refuse to ignore the possibility that cases could be overlooked:

“[When asked about the possibility of elimination by 2020] That is a firm no. We do not yet know how to reach it because, so far, we only have coverage of the population at high risk. We know that currently with the WHO we have categorized the high-risk, medium-risk and low-risk areas, but when we take the areas of active screening together, we do not go well beyond 20% of the population.”

(9, management)

All our respondents identified coverage as a core issue, “if we don’t put the emphasis on coverage, the other ingredients will not kick in” (19, management) explained a manager. The suggestion is to reschedule activities and mobile units to villages not visited in some time (the so-called *reprogrammation*), and put resources “village by village, then we will know whether the disease has been eradicated” (21, mobile unit).

The second objection that was raised to elimination by 2020 is the lack of emphasis on the vector in current efforts: “the strain [trypanosomiasis parasite] must be destroyed, if the tsetse fly continues to multiply, to occur, it is difficult to believe that we can have a total elimination” (13, frontline non-mobile). Half the respondents insisted on the need to engage more substantially with the vector, pointing out to challenges in the current interventions.

“Secondly, in this same population, you will try to set traps wherever there are tsetse flies. We educate them [the population] but these same traps are stolen by the same population […], even if you designate people to monitor these traps and catch tsetse flies […] they ask for money, they must be motivated.”

(7, mobile unit)

Overall, we see that HAT frontline workers are not as convinced as international actors of the possibility of an imminent elimination of the disease. Based on their experience and practice of HAT control, they explain that such elimination would imply two hard to achieve premises: that PNLTHA activities stretch to all the areas potentially affected by HAT and that the vector is better controlled (they do not explain what this would mean in practice, but agree that not all flies can be eradicated). It is also clear that frontline workers do not see a clear-cut distinction between the elimination and the eradication targets.

### 4.3. Personal Effects of Elimination

Bearing in mind that many frontline workers do not see elimination as imminent, let’s now turn to the workers’ perspectives on the effects that elimination would have on them, as individuals.

When asked about their feelings towards the idea of elimination, frontline workers at all levels of responsibility first expressed keen enthusiasm rather than fear for their livelihoods: “I am not worried, I rejoice” explained a mobile unit worker (18, management). The emotional commitment of the HAT workers explained in Section 4.1 should not be underestimated: the interviewees shared a sense of being part of a mission, which is the elimination of the disease, and all personally witnessed the burden of HAT. The primary emotion associated with the possible elimination of HAT was pride, and it was echoed by every research participant: “our objective is not to maintain HAT but to get it out of our country, and if we can eliminate it, that is a source of pride for us. And for our lives” (7, mobile unit).

When seeking to push further on the question of what would happen if the elimination became a reality, different attitudes prevail, sometimes expressed one after the other. The most common was for the frontline workers to mention their status as civil servants and explain that they trust the state to re-allocate them to relevant posts: “the state cannot abandon these people, it will introduce them to the general hospital to treat certain diseases, even in health zones” (16, frontline non-mobile). It is implicitly assumed that it would probably mean a lower level of income: “as we are agents of the state, if the state could pay civil servants properly, we can continue to survive, it is not the end of the world” (5, mobile unit), and the older generations were confident that they would probably not be re-allocated but rather retire with “honourable retirement benefits”. In many cases, because the possibility of elimination of HAT is not entertained as a genuine imminent possibility, the sense is that HAT expertise will continue to be needed even if the number of cases is very low, but the work would be slightly different, more related to passive screening:

“The people in the mobile units are not afraid; they say that the work will not end because the disease will not end. […even if] the programme (PNLTHA) tends towards the end; the disease will not end; it is since the creation”

(16, frontline non-mobile)

Integration in the non-HAT health system is then seen as the main option for many, who also display a sense of confidence in their skills and abilities. It is often doubled with a strong belief in God’s will or in one’s personal capacity to find an alternative livelihood.

Not all frontline workers are in the same situation, though. Fear is more visible among those who are less qualified or newer and are often not registered on the national level books [*non-mécanisé*], meaning that they were hired locally, or that they are, sometimes at the same time, not on the official payroll [*non-payé*]. Even with them, though, a strong sense of resilience seems to prevail:

“I am in a new unit without a personnel number [unregistered, *non-mécanisé*], that’s something that scares me; I can become unemployed in that sense. But I know that whoever gave me this job knows how he’s going to use me. If he is tired of me, I will do agriculture; I will fish if I still have the strength.”

(2, mobile unit)

“I’m a registered and ‘unpaid’ [by the Ministry of Health, *non-payé*] employee, but we’ll always go to the inspection [provincial health authorities], or we’ll manage. There is always life.”

(1, mobile unit)

Such a form of (anticipated) resilience is not uncommon in DR Congo, a country which has endured decades of instability and violence. This is not to say that losing the HAT premiums or HAT-related job is not a concern:

“[…] it worries us; it must worry us because where you were able to afford school fees for children, you are stuck with that. […] As you are given something that is not sufficient enough, you try to find a way, but then you will get used to it.”

(15, frontline non-mobile)

Managers, even if they share the views that elimination may not happen too soon, have nevertheless been thinking about alternative options for their staff:

“The majority of our service providers, our employees, they are under-qualified, and they will have a lot of difficulties to learn other knowledge. We have a vast project for them that will involve training people, obtaining scholarships […], on-the-job training for everyone, […] not specific to sleeping sickness. […]. And thirdly, we believe that for our staff, as many of them, we must obtain the *mecanisé* status [have them on the ministry books].”

(10, management)

Beyond thinking about possible strategies, managers have already had their laboratory technicians and nurses undergo training on malaria, filariasis, and onchocerciasis. As a manager pointed out, “if sleeping sickness is over today, filariasis will not leave today, what would prevent us from continuing to fight communicable diseases with the same teams?” (20, mobile unit). In addition to this, active networking and positioning by staff seem to be taking place as well, as explained by a mobile unit leader:

“We always ask the chief doctors of the zones whether it is the vaccination period and whether they can take people from the mobile units so that they can also immerse themselves in other stories [other contexts] so that when the time comes, they will still be useful to Congolese society.”

(18, management)

Overall, HAT frontline workers appear to have ambiguous attitudes towards their future in a post-HAT DR Congo. All of them appear genuinely delighted to imagine that such a world may exist, despite the implications it may have for their livelihoods The more experienced and senior workers appear confident that as civil servants, they will be allocated to a new position—which they believe they are qualified and competent for because of their training. Less secure workers seem aware that the risk of unemployment is higher for them but are also confident in their resilience capacity. The sub-section has also shown that managers are already deploying strategies for providing their staff with the best chances of securing alternative positions or livelihoods.

## 5. Discussion

The interviews reveal frontline workers who form a community of experienced HAT experts who are genuinely dedicated to the elimination of the disease. As it was documented in other cases, their experience is also the local historical memory of HAT research and programmes in the area [34]. The frontline workers form a crucial resource that is trusted by the population and recognised for its capacity. Their value should not be underestimated when operating in the difficult context of the DR Congo; this is for implementing HAT-related innovations, understanding the progress that is being made in the field, but also strengthening the health system in general.

The findings need to be considered in the context of the recent evolution of efforts to fight HAT in the DR Congo, bearing in mind that the mobile units are the ones that feel the most pressure due to the anticipated replacement of mobile units with mini-mobile units and changes in the micro-planification of activities. Overall, the interviewees show some scepticism towards the possibility of a very near elimination of HAT in the DR Congo. It is useful to note that it is only their perspective (and not the perspective of the PNLTHA as a whole), which is informed by their (partial) field experience and work rather than a comprehensive picture of the situation in the whole of DR Congo. On the ground, those working on control think more in terms of the absolute number of cases (that they see) than in terms of incidence rates. There might also be some confusion with the word ‘elimination’, which is not always used consistently by policymakers and academics alike [35]. The interviewees understand elimination as the elimination of the disease, by which they mean a reduction to zero of the HAT incidence, which is different from WHO’s idea of elimination of HAT as a public health problem that still requires “measures to prevent the re-establishment of transmission” [35]. Some of the interviewees also seem to conflate elimination with eradication, which is the situation when no intervention whatsoever is required any longer. Beyond the question of the definition, the issues pointed out by the research participants is that the perceived lack of control of the vector (which was recently reinforced on over 120,000 km^2^ with the support of the Liverpool School of Tropical Medicine) and the ‘blind spots’—the areas without active screening—are impediments to elimination. Here again, it is useful to remind that the reality seen from the field does not necessarily include a broader level vision of recent efforts to increase data quality, which include projects funded by the Bill and Melinda Gates Foundation and Belgian government since 2015 in the provinces of Kwangu, Maindombe and Kwilu. This caveat aside, the perspectives of the frontline workers align with research pointing to the necessity to properly combine screening, treatment of patients, and vector control to eliminate HAT rapidly [31,36].

The frontline workers also express a discourse that is not necessarily always visible in the policy declarations and academic literature that keenly emphasise technological progress: eliminating HAT requires, in their view, a deeper investment in detection and treatment. They partially echo research that discusses the merits of passive screening [9,26] and suggests difficulties in spreading efficient and easy detection tools such as the combination of RDT with loop-mediated isothermal amplification (LAMP) [5], not so much because it is technically hard to detect cases but rather because of the epidemiological context and the lack of HAT skills in the system (outside PNLTHA) [36]―in other words, the strength of the health system. As one of the interviewees put it: “it is more a question of organisation than just tools”.

The changing nature of the work of the frontline health workers, and in particular the difficulties they face enrolling people into active screening [26] and collaborating with the local health authorities [10], points to another set of issues that are not discussed in great length in the literature on HAT elimination. Two dynamics seem to be reinforcing each other. On the one hand, as in all cases of disease elimination, the fact that the disease becomes rarer means that people and their leaders pay less attention to it and are less interested in investing resources in it or endure hardship to fight it. This is similar to what was observed with polio and smallpox campaigns [37,38,39]. On the other hand, the endemic regions of DR Congo also witnessed profound socio-political changes in recent decades and gaining the trust of communities and their leaders is, according to our findings, harder than before (regardless of the motive). Palmer and colleagues also touch on the issue in a paper on Uganda [40]. The context is slightly different as they look at refugees, but the similarities are clear: populations are seen a ‘belonging’ to authorities who are their unavoidable gatekeepers, and relations with them seem increasingly monetised. This must also be considered against the background of decades of international aid and changing incentive structures, including the ‘culture of per diem’ [41,42] that makes it harder for PNLTHA teams to obtain support from community leaders and even health workers without providing monetary incentives. Again, we know from other elimination campaigns that community involvement is absolutely central to elimination [39]. The combination of the difficulty of enrolling people in screening and the difficulty of collaborating with local politico-administrative authorities should be fully considered as it may have an influence on the actual cost [43] and efforts needed to reach elimination.

In answer to our second research question, we found that frontline workers display ambivalent attitudes towards their post-elimination future. Many interviewees doubt that elimination will arise too soon and also appear confident that as civil servants, they will simply be redeployed to other services. Staff of mobile team units, who also have in mind ongoing changes in the organisation of active screening, are probably the most concerned of the interviewees. Polio and smallpox eradication campaigns around the world suggest that such redeployment may happen [13], but the case of HAT in DR Congo and Bandundu may be more complicated for two reasons. First, the level of verticalization or ‘siloisation’ is very high [44] and workers are very specialised. Contrary to other countries, many HAT frontline nurses and lab technicians mostly, when not only, work on HAT and may not have much experience working on other medical conditions. Managers seem mindful of this issue; they have taken measures to facilitate such redeployment when and if it is needed, for instance they organised the temporary placement of their staff in other units (e.g., in vaccination campaigns), so they gain experience and can demonstrate their usefulness. They also organised refresher courses on non-HAT health issues. Other options are available but were not directly mentioned by the mobile teams and frontline workers; they include maintaining the mobile teams but allocating them to other tasks such as population census, curative healthcare activities (with the mobile unit become a de facto more generalist mobile clinic) in remote areas, or the coordination of preventative healthcare efforts. The second reason redeployment may be challenging is that some frontline workers are hired locally and do not appear in the central level registries. As in other fields of the public service in DR Congo [45], a higher level of struggle is anticipated for those workers.

Beyond the question of the resilience of HAT frontline workers to post-HAT changes in the labour market, a more immediate question is suggested by the research participants. They point out that their expertise may still be useful for quite some time after a ‘zero new cases’ situation is declared, for monitoring the situation as the local, non-specialised workforce, is not seen as able to do that—an argument that echoes the need for keeping human expertise during the final stages of elimination [36,46]. Such integration, from the perspective of human resources and human expertise rather than tools (e.g., rapid diagnostic tests) and activities, is not really discussed in the emerging literature on integration [24,47]. Future research should explore scenarios of integration that do not only see health facilities as covering new aspects of healthcare but also as integrating practitioners with specific experience. The issue may be DR Congo (or even Bandundu) specific―other countries have a much smaller specialised HAT workforce―but it is worth considering given the place of DR Congo in the HAT epidemic.

There are clear limitations to the present study: it is limited to a few sites, it only collects the views of a few individuals (even if saturation seems reached among them), and it does not address at all issues such as the gendered perspective on HAT (all respondents, as most of the HAT workforce, is male). The article should be considered a preliminary effort towards better understanding frontline realities and livelihoods rather than definitive work on the topic. It is also important to state the exceptionalism of DRC, and especially Bandundu, compared to other contexts: it has high and continuous levels of disease, a culture of incentives, and a history of centralisation of HAT expertise (this is different from many other settings when HAT coordinators are primarily interested in exposing generally skilled health workers to HAT [26]). Replicating the research in other sites, and especially sites that are more peripheral to the HAT elimination efforts would be useful to understand the situation at a more advanced stage of elimination, as would a more systematic review of the concerns expressed by the respondents and in particular communities’ and leaders’ changing attitude towards HAT and the health system in general and the risks associated with a focus of active screening on only a selected priority endemic areas.

## 6. Conclusions

The perspectives of frontline workers cast light on aspects of HAT elimination that are not at the forefront of many academic and policy publications: the growing socio-political difficulty of active screening, field perspectives on the coverage of detection and treatment activities, and the strong community of practitioners around HAT. The findings shift the perspective from an emphasis on innovations (drugs, tests, vector targets) to the need to pay sustained attention to the organisation of HAT elimination. While the findings nuance the possibility of an imminent elimination of HAT, they also suggest concrete pathways to improving the efficacy of elimination efforts.

## Figures and Tables

**Table 1:** Profiles of the research participants.

Category	Role	Gender	Experience	Interview Number
mobile unit	2 Community outreach workers 1 Secretary 2 Technicians (lab) 6 Leaders (nurse/technician, *chef d’équipe mobile*)	all males	28 years (SD: 10.9)	1, 2 17 20, 21 3–8
Frontline non-mobile	4 HAT centre (CDTC) chief nurse 2 HAT hospital centre chief nurse	all males	25.8 (6.37)	11–14 15–16
management	2 HAT provincial management (supervisor) 2 HAT provincial management (coordinator)	all males	31 (9.89) 11.5 (2.12)	18, 19 9, 10

## Appendix A. Interview Guide

- Could you briefly tell us about who you are, how and when you started working in sleeping sickness?
- What have been your main responsibilities in the fight against sleeping sickness so far?
- In your role you visit several places where there is sleeping sickness, considering its current evolution, do you think that we will eliminate sleeping sickness
- Currently we are talking about the elimination of the HAT by 2020, do you think this objective is achievable in the DRC?
- What does it mean for you to be on the ground when it comes to the elimination of sleeping sickness in the DRC?
- Some interviewees say that innovations in HAT treatment, diagnosis and new screening approaches are needed to eliminate sleeping sickness. Others, on the other hand, believe that even if there are innovations if the coverage of the population at risk remains low, elimination will not be possible. What are you saying?
- Some interviewees say that you members of the mobile teams and HAT treatment centre are afraid of the elimination of sleeping sickness for fear of losing your job when the disease disappears?
- If tomorrow sleeping sickness were to disappear, what would you do?
- Do you control diseases other than HAT? Is there anything else your team could do that you think your team could do?
- In your opinion, what are the major challenges to eliminating sleeping sickness in the DRC?
